# Evaluation of Local Skeletal Muscle Blood Flow in Manipulative Therapy by Diffuse Correlation Spectroscopy

**DOI:** 10.3389/fbioe.2021.800051

**Published:** 2022-01-11

**Authors:** Yasuhiro Matsuda, Mikie Nakabayashi, Tatsuya Suzuki, Sinan Zhang, Masashi Ichinose, Yumie Ono

**Affiliations:** ^1^ Electrical Engineering Program, Graduate School of Science and Technology, Meiji University, Kawasaki, Japan; ^2^ Faculty of Medical Science, Nippon Sport Science University, Yokohama, Japan; ^3^ Japan Society for the Promotion of Science (JSPS), Tokyo, Japan; ^4^ Department of Electronics and Bioinformatics, School of Science and Technology, Meiji University, Kawasaki, Japan; ^5^ Human Integrative Physiology Laboratory, School of Business Administration, Meiji University, Tokyo, Japan

**Keywords:** diffuse correlation spectroscopy, heart rate variability, manipulative therapy, muscle blood flow, muscle stiffness, vascular conductance

## Abstract

Manipulative therapy (MT) is applied to motor organs through a therapist’s hands. Although MT has been utilized in various medical treatments based on its potential role for increasing the blood flow to the local muscle, a quantitative validation of local muscle blood flow in MT remains challenging due to the lack of appropriate bedside evaluation techniques. Therefore, we investigated changes in the local blood flow to the muscle undergoing MT by employing diffuse correlation spectroscopy, a portable and emerging optical measurement technology that non-invasively measures blood flow in deep tissues. This study investigated the changes in blood flow, heart rate, blood pressure, and autonomic nervous activity in the trapezius muscle through MT application in 30 volunteers without neck and shoulder injury. Five minutes of MT significantly increased the median local blood flow relative to that of the pre-MT period (*p* < 0.05). The post-MT local blood flow increase was significantly higher in the MT condition than in the control condition, where participants remained still without receiving MT for the same time (*p* < 0.05). However, MT did not affect the heart rate, blood pressure, or cardiac autonomic nervous activity. The post-MT increase in muscle blood flow was significantly higher in the participants with muscle stiffness in the neck and shoulder regions than in those without (*p* < 0.05). These results suggest that MT could increase the local blood flow to the target skeletal muscle, with minimal effects on systemic circulatory function.

## Introduction

Manipulative therapy (MT) is a traditional procedure applied to the musculoskeletal system as part of a medical treatment, sports conditioning, and health promotion. MT techniques include kneading, rubbing, tapping, and pushing performed by a practitioner’s hands (Goats, 1994; Çetkin et al., 2019). The demand for MT has rapidly increased during the coronavirus disease 2019 pandemic, due to the excessive tension, pain, and discomfort of the skeletal muscles from the excessive use of computers and hand-held devices (Ishikawa et al., 2017; Kramer and Kramer, 2020; Lin et al., 2020).

Although MT has been recognized as a non-invasive and cost-effective treatment for relieving muscle tension and pain (Driessen et al., 2012; Domingo et al., 2017), clinical evidence supporting MT remains scarce compared to other alternative medical procedures (Tiidus, 1997). Furthermore, findings on the presence or absence of an MT-induced increase in local blood flow, which is one of the primary effects expected from MT, are inconsistent.

The lack of accurate and practical bedside techniques to quantify deep-tissue blood flow could explain this inconsistency. Several techniques, such as the thermodilution method (Reuter et al., 2010), Xenon clearance method (Thomas et al., 1979), and positron emission tomography (Grafton et al., 1992) have been utilized to quantitatively measure tissue blood flow. However, these invasive and semi-invasive techniques are inappropriate for investigating the therapeutic effect of MT, which mainly targets patients and/or elderly individuals with physical decline. Perfusion MRI techniques, which offer minimally or non-invasive measures of blood flow in the skeletal muscle (Wigmore et al., 2004; Englund and Langham, 2020), are also unsuitable for monitoring the therapeutic effect of MT, as it requires physical manipulation. The ultrasound Doppler method is another non-invasive technique for measuring tissue blood flow (Saltin et al., 1998; Kitano et al., 2005; Ichinose et al., 2021). However, changes in tissue microcirculation from locally applied MT might not be captured with the ultrasound Doppler method, which detects the total blood flow of the conduit vessels connected to the whole limb. Although extensively employed to investigate the effect of MT, none of these methods could identify MT-related increases in blood flow (Tiidus and Shoemaker, 1995; Shoemaker et al., 1997; Hinds et al., 2004). Interestingly, there are reports of locally increased blood flow, which could explain the elevated cutaneous and/or intramuscular temperature at the site of MT application (Drust et al., 2003; Sefton et al., 2010; Portillo-Soto et al., 2014; Monteiro Rodrigues et al., 2020).

A novel optical approach of diffuse correlation spectroscopy (DCS) was used to overcome these methodological limitations. DCS provides information on the microvascular blood flow through changes in the statistical properties of the reflected intensity of near-infrared light caused by the movement of red blood cells in the tissue (Yu et al., 2005; Durduran et al., 2010). Studies have validated the significant correlation between DCS and other established techniques, such as arterial spin-labeled perfusion MRI (Yu et al., 2007; Carp et al., 2010) and fluorescent microsphere techniques (Zhou et al., 2009) in the detected blood flow indices. Previous studies combining DCS measurements of different inter-probe distances and skin perfusion measurements under various physiological maneuvers have confirmed the capability of DCS to capture blood flow changes in the skin and muscle tissues (Nakabayashi and Ono, 2017; Ichinose et al., 2018; Bartlett et al., 2021). The portable and relatively low-cost experimental setup could also benefit the future clinical application of DCS for bedside blood flow monitoring (Yu, 2012).

This study investigated the effect of MT on the local and systemic circulatory responses in participants with and without muscle stiffness. In addition to the local blood flow measured by DCS, the heart rate (HR), cardiac autonomic nervous tone, and arterial blood pressure were simultaneously monitored as indices of systemic circulatory response (Diego et al., 2004; Kaye et al., 2008; Diego and Field, 2009). Our hypotheses were twofold: First, MT could increase local blood flow in the targeted muscle compared to the same time of rest. Second, muscle stiffness may be caused by insufficient blood supply, and could thus benefit from the therapeutic effects of MT.

## Materials and Methods

### Participants

A total of 30 healthy volunteers (13 men and 17 women; 19–55 years; mean age, 29.5 ± 10.4 years) without neck and shoulder injury participated in the study. All participants received sufficient explanations of the experimental procedures and provided written informed consent. The study was performed according to the Declaration of Helsinki and was approved by the Ethical Review Committee of the School of Science and Technology, Meiji University (approval number: 19-538).

### Muscle Blood Flow, Systemic Blood Pressure, and HR Recordings

**FIGURE 1:**
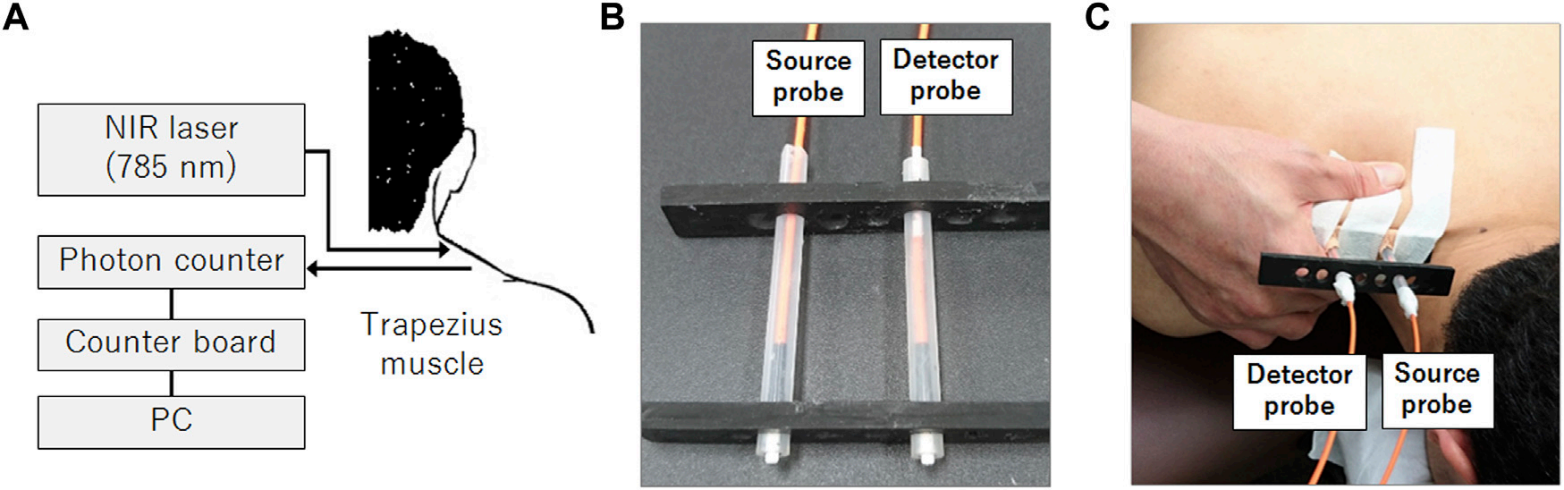
DCS system configuration for blood flow measurement in the muscle. The DCS system consisted of a 785-nm near-infrared light source and a single photon counter **(A)**. Source and detector optical fibers are housed in a rubber holder **(B)** and attached to the skin surface using medical adhesive tapes above the right trapezius muscle **(C)**. These optical fibers deliver near-infrared light to the skin surface (source probe) and detect the light scattered through the tissue (detector probe). The therapist applied manipulative therapy on the towel placed over the shoulder during the experiment, but the towel was removed in the photograph to indicate the arrangement of the hand and the probes during the intervention.

Room temperature was maintained at ∼26°C throughout the experiment. Simultaneous measurement of muscle blood flow, systemic blood pressure, and electrocardiogram (ECG) was performed when participants were in a prone position. Optical probes of the DCS system developed in-house (Nakabayashi and Ono, 2017; Ichinose et al., 2018; Nakabayashi et al., 2018; Ono et al., 2018, Ichinose et al., 2019, 2021) were attached to the skin surface just above the superior fibers of the right trapezius muscle at the center of the line, connecting the spinous process of the seventh cervical vertebra and the acromion of the right scapula to monitor the local blood flow (Figure 1). The optical probes were secured on the skin surface with a medical adhesive tape (see Supplementary Video S1 for a more detailed fixation procedure of the optical probes). The DCS system has been thoroughly described in previous studies (Boas et al., 1995; Durduran et al., 2010; Nakabayashi and Ono, 2017; Ono et al., 2018). The system consisted of a long coherent, continuous near-infrared light source (DL785-100-SO, Crysta Laser, NV, United States) and a single photon-counting device (COUNT-T-100FC, LASER COMPONENTS, Germany). The emitted light was guided to the skin surface using a multi-mode optical probe (FT400EMT, Thorlabs Japan Inc., Japan), and the scattered light was guided to the photon-counting device through a single-mode optical probe (S630-HP, Thorlabs Japan Inc., Japan) (Figures 1B,C). The approximate depth of the DCS measurement below the skin surface is considered one-third to one-half of the distance between the emitter and detector optical probes (Patterson et al., 1995; Yu et al., 2005), which was 3 cm in this study (Figure 1). Since the distance from the skin surface to the trapezius muscle layer in adults is reported to be 4.9–6.5 mm (Flodgren et al., 2006; Sandberg et al., 2007), the blood flow information could reliably reflect blood flow changes in the trapezius muscle. The blood flow index (BFI), a relative value equivalent to the mean blood flow within a tissue volume through which the light diffuses, was calculated at a sampling rate of 1 Hz throughout the experiment using an in-house developed software correlator (Dong et al., 2012) implemented in LabVIEW and MATLAB. Experiments were performed in a dark room to prevent extraneous light from contaminating the DCS signal to the detector probe.

The pneumatic cuff of a sphygmomanometer (Tango M2 Stress Test Monitor, Sun Tech Medical Inc., NC, United States) was wrapped around the left upper arm to measure the systemic blood pressure every 1 min. Electrodes were attached to the right wrist and the left ankle to measure ECG in the lead II configuration at a sampling rate of 1,200 Hz using a bio-amplifier (g.USBamp, g.tec medical engineering GmbH, Austria).

### Experimental Procedures

A licensed therapist with >20 years of clinical experience [Y. M.; Japanese national qualification of Judo therapy (World Health Organization, 2001; Nishikitani et al., 2008)] evaluated the participants’ stiffness of the trapezius muscle on arrival to the laboratory. The reliability of the palpation by a trained therapist for localizing the regions of muscle stiffness was previously confirmed (Sciotti et al., 2001; Barbero et al., 2012). Participants were diagnosed with muscle stiffness if they complained of painful and stiff muscles in the neck and shoulders on the experimental day, and if the therapist found recognizable muscle stiffness at the measurement site by palpation. Even when the participant complained of pain or stiffness in the muscles other than the measurement site, they were classified as muscle stiffness negative group if the therapist did not recognize muscle stiffness at the measurement site where MT was applied. Twelve participants (12 women; 20–55 years; mean age, 32.6 ± 12.1 years) met these criteria and were classified into the muscle stiffness positive group [ST (+)]. The other 18 participants (13 men and 5 women; 19–47 years; mean age, 27.4 ± 8.5 years) were included in the muscle stiffness negative group [ST (−)].

**FIGURE 2:**
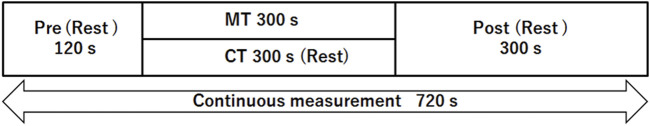
Measurement protocol. Participants received manipulative therapy (MT) for 300 s (intervention period) in the MT condition, whereas they remained still on the measurement table during the control condition (CT). Pre-and post-rest periods were identical between conditions. Local blood flow of the trapezius muscle, systemic blood pressure, and electrocardiogram were continuously measured throughout the experiment.

All participants underwent two sessions of blood flow measurements in a fixed order of control (CT) followed by MT. The order of conditions was not randomized to prevent the aftereffect of MT on the local blood flow and other physiological measures. Each session consisted of 120 s of pre-intervention rest (pre), followed by 300 s of intervention and another 300 s of post-intervention rest (post) (Figure 2). In the CT condition, participants remained on the measurement table throughout the intervention without any treatment. Conversely, in the MT condition, the therapist applied MT to the measurement site on the right trapezius muscle using the kneading method, wherein physical stimulation could be applied to the target muscle without contacting the therapist’s hand with the optical probes and the probe holder. In the kneading method, the therapist lightly squeezes the muscles by grasping the muscle between the pads of the thumb and four fingers from the lateral side at ∼2 s per cycle (see Supplementary Video S2 for a demonstration of the kneading method used in this study). Conforming to the traditional style of Judo therapy, the therapist applied MT through a towel placed over the shoulder. The placement of the towel also helped minimize thermal stimulation from the therapist’s hand which may affect the local blood flow. The frequency of manipulation was ∼25–30 times/min regardless of the presence or absence of muscle stiffness. MT strength was adjusted to avoid exceeding the individual pain threshold. After the experiment, the probe position was confirmed to be maintained in the original position for each participant (see Supplementary Video S3 for the stability of probe positions after 300 s of kneading).

### Data Analysis

**FIGURE 3:**
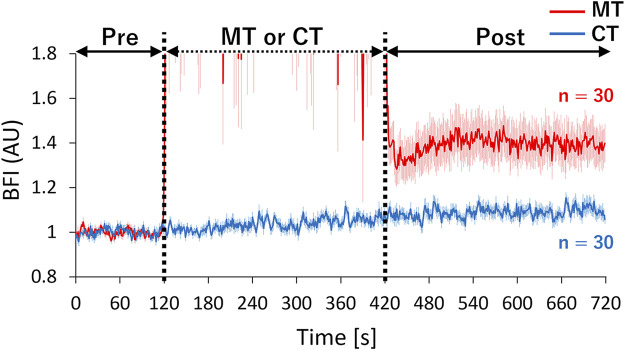
Time-course changes of mean normalized BFI in CT and MT conditions. The therapist performed MT for 120–420 s in the MT condition. BFI values were normalized based on the mean value during the pre-period. Relative increases in the local blood flow were compared between conditions by superimposing the normalized BFI time-courses. Solid lines show mean values, and shaded areas show standard errors. BFI: blood flow index, Red line: MT condition, Blue line: CT condition, AU: arbitrary units.

The mean BFI values, systolic arterial pressure, mean arterial pressure (MAP), diastolic arterial pressure, HR, and vascular conductance index (VCI: BFI/MAP) during the pre-and post-periods were determined in each participant and subjected to statistical comparisons between conditions. We excluded data during the intervention period from the analysis due to the presence of significant motion artifacts during MT. The first 60 s of the post-period were also excluded from the analysis, as the BFI showed transient changes during that time (Figure 3). The time course of BFI values was further normalized by the mean BFI values during the pre-period to determine the relative increase in blood flow after each condition.

In addition, we evaluated the autonomic nervous activity using HR variability (HRV), resulting from the analysis of ECG data. The beat-to-beat RR interval data were detected and resampled at 60 Hz, and a time-frequency analysis was performed using Morlet wavelets. We determined the integrated spectral power of the high-frequency (HF, 0.15–0.4 Hz) and low-frequency (LF, 0.04–0.15 Hz) components. HF and LF/HF were used as indices of parasympathetic and sympathetic activity, respectively (Aluwi et al., 2016; Laborde et al., 2017; Task Force of the European Society of Cardiology, and the North American Society of Pacing and Electrophysiology, 1996). The data of one participant from the ST (−) group contained a large amount of noise in the ECG, and thus were excluded from the HRV analysis.

To further investigate the physiological characteristics of muscle stiffness and the effect of MT on ameliorating such, the BFI, MAP, and VCI in the MT condition were compared between the ST (+) and ST (−) groups. We further calculated changes in the BFI after the MT intervention [∆BFI: (mean BFI in the post period)—(mean BFI in the pre period)] from the BFI data acquired in the MT condition. The mean BFI in the pre-period (baseline BFI) and ∆BFI values were compared between the ST (+) and ST (−) groups, and the correlation between these two indices was investigated. If the baseline BFI of the trapezius muscle was smaller in ST (+) participants than in ST (−) participants, decreased blood flow to the target muscle could be identified as the potential phenotype of muscle stiffness. If the ∆BFI is larger in ST (+) participants than in ST (−) participants, increased blood flow could be considered a therapeutic effect of MT which facilitates local circulation.

The changes in BFI between the pre-and post-MT periods, baseline BFI, and ∆BFI values were additionally compared between the subgroups of female participants from the two groups [*n* = 12 and 5 in groups ST (+) and ST (−), respectively] to investigate the effect of MT when controlling for sex differences in the participants from the original groups. We also tested the correlation between baseline BFI and ∆BFI values within each group only for female participants.

We applied the non-parametric Wilcoxon signed-rank test to compare the values between the pre- and post-periods in the same condition and between conditions in the corresponding periods. The Mann-Whitney *U* test was used to compare the values between the ST (+) and ST (−) groups. A Bonferroni correction for multiple comparisons was applied when necessary, and Spearman’s rank correlation was calculated to test the relationship between the baseline BFI and ∆BFI in each group. Statistical significance was set at *p* < 0.05.

## Results

The changes in the BFI, normalized BFI, blood pressure, HR, cardiac autonomic indices, and VCI values before and after the CT and MT interventions are summarized in Table 1. The BFI and normalized BFI were significantly increased in the post-compared to the pre-period in both MT and CT conditions. In addition, the post-intervention values in the MT condition were significantly larger than those in the CT condition. There were no significant differences in blood pressure, HR, and cardiac autonomic indices between the pre-and post-periods in the same conditions and between the corresponding periods of the two conditions (CT and MT). Accordingly, the VCI was significantly increased in the post-compared to that in the pre-period for the MT and CT conditions. In addition, the post-intervention VCI in the MT condition was also significantly larger than that in the CT condition.

**TABLE 1:** Changes in blood flow, blood pressure, heart rate, cardiac autonomic indices, and vascular conductance index before and after manipulative therapy.

	MT		CT	
	Pre	Post	Pre	Post
BFI [× 10^−9^ cm^2^/s]	4.8 (4.2, 5.7)	5.8 (5.1, 7.8)*^,†^	4.4 (3.5, 5.2)	4.8 (3.9, 5.6)*
Normalized BFI	1	1.2 (1.0, 1.6)*^,†^	1	1.1 (1.0, 1.1)*
SAP [mmHg]	111 (104, 122)	111 (104, 120)	114 (105, 122)	115 (104, 121)
MAP [mmHg]	80 (77, 89)	80 (76, 87)	83 (77, 91)	82 (77, 88)
DAP [mmHg]	67 (62, 74)	65 (60, 72)	68 (64, 75)	66 (62, 73)
HR [bpm]	67 (59, 71)	65 (60, 74)	65 (59, 73)	65 (58, 74)
HF [10^−3^ ms^2^]	3.9 (1.8, 8.5)	3.7 (2.0, 10.9)	2.8 (1.7, 8.6)	3.7 (1.7, 8.7)
LF/HF	0.3 (0.2, 0.8)	0.4 (0.2, 0.7)	0.3 (0.2, 0.6)	0.3 (0.2, 0.7)
VCI [× 10^−11^ cm^2^/(s mmHg)]	6.3 (5.5, 7.3)	6.9 (6.1, 11.6)*^,†^	5.4 (4.3, 6.8)	6.0 (4.9, 6.8)*

Data are presented as the median (first quartile, third quartile). Asterisks (*) indicate significant increases (*p* < 0.05) at the post-period relative to the pre-period. A dagger (†) indicates a significant increase (*p* < 0.05) relative to the CT condition in the post period. MT: manipulative therapy condition; CT: resting condition; BFI: blood flow index; normalized BFI: normalized blood flow index; SAP: systolic arterial pressure; MAP: mean arterial pressure; DAP: diastolic arterial pressure; HR: heart rate; HF: high-frequency component of heart rate variability; LF/HF: ratio of low to high-frequency components of heart rate variability; VCI: vascular conductance index.

The median BFI values showed a gradual increase along with the measurement order (CT pre < CT post ≈ MT pre < MT post), suggesting the possibility that the post-intervention BFI increase found in both conditions might originate from the elapsed time while lying on the measurement table irrespective of intervention type. Therefore, we further investigated the relative increase in BFI from baseline in both conditions (Figure 3). The waveform during the intervention in the MT condition was excluded due to motion artifacts. The mean normalized BFI values of the post-period in the MT condition increased by ∼40% compared to the pre-period. However, the increase in BFI in the post-period remained at ∼9% compared to the pre-period in the CT condition. These results also confirmed that MT significantly increased the blood flow in the target muscle.

The MT-related changes in BFI, normalized BFI, MAP, and VCI in the subgroups with or without muscle stiffness in the measurement site are shown in Table 2. The post-MT BFI and normalized BFI were significantly larger in the ST (+) group than in the ST (−) group. Comparing only the female participants between the two groups, the presence and absence of a post-MT increase in BFI in the ST (+) and ST (−) groups were also confirmed (*p* = 0.003 and 0.563 with Bonferroni correction, respectively). MAP was not significantly different within and between groups. The VCI of the ST (+) group increased significantly after MT.

**TABLE 2:** Changes in the blood flow, mean arterial pressure, and vascular conductance index before and after MT in the subgroups with or without muscle stiffness in the neck and shoulder region.

	Muscle stiffness positive		Muscle stiffness negative	
	Pre	Post	Pre	Post
BFI [× 10^−9^ cm^2^/s]	4.7 (3.8, 5.6)	7.7 (5.9, 9.9)*^,†^	4.8 (4.3, 6.0)	5.2 (4.8, 6.4)
Normalized BFI	1	1.7 (1.4, 2.1)*^,†^	1	1.1 (1.0, 1.2)
MAP [mmHg]	78 (75, 94)	77 (73, 95)	87 (80, 90)	83 (78, 88)
VCI [× 10^−11^ cm^2^/(s mmHg)]	6.2 (4.0, 7.2)	9.9 (7.2, 12.8)*	6.3 (5.5, 7.1)	6.5 (5.8, 7.1)

Data are presented as the median (first quartile, third quartile). Asterisks (*) indicate significant increases (*p* < 0.05) at post-relative to pre-period. A dagger (†) indicates a significant increase (*p* < 0.05) relative to the muscle stiffness negative group in the post-period. Muscle stiffness positive: ST (+) group; muscle stiffness negative: ST (−) group; BFI: blood flow index; normalized BFI: normalized blood flow index; MAP: mean arterial pressure; VCI: vascular conductance index.

**FIGURE 4:**
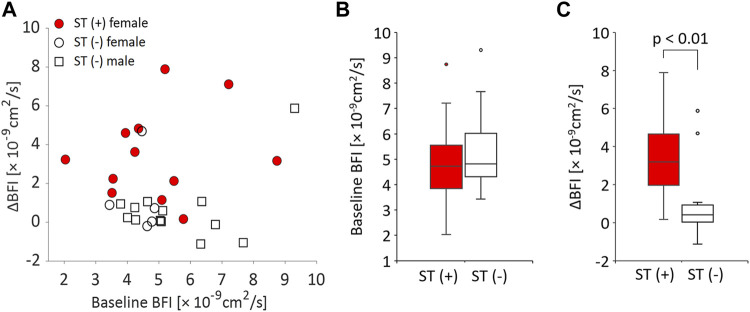
Relationships between baseline BFI and MT-related BFI increase (ΔBFI) in participants with and without muscle stiffness. **(A)** Scatter plot of baseline BFI and ΔBFI for all participants. Each circle and square represent a female or male participant, respectively. Participants with muscle stiffness are shown by filled circles [ST (+): female]. Participants without muscle stiffness are depicted by open circles [ST (−): female] or open squares [ST (−): male]. **(B)** Box plot of the baseline (pre) BFI in the ST (+) and ST (−) groups. **(C)** Box plot of ΔBFI in the ST (+) and ST (−) groups.

Since these results demonstrated varied microcirculatory responses to MT depending on the individual status of muscle stiffness, the relationship between the baseline BFI and the increase in BFI after MT was further investigated (Figure 4). The distribution of the baseline BFI was comparable between participants with and without muscle stiffness (Figures 4A,B), whereas the post-MT increase in BFI was significantly larger in participants with muscle stiffness (Figures 4A,C), indicating that MT had a greater impact on the increase in local muscle blood flow in muscles with stiffness relative to those without. The relative median increase in post-MT BFI was 64 and 7% in the ST (+) and ST (−) groups, respectively. The comparable baseline BFI between groups and the significant increase in the post-MT BFI with ST (+) over ST (−) group were also confirmed when only female participants were analyzed. There was no significant relationship between the baseline BFI and post-MT increase in BFI in any of the groups tested.

## Discussion

This study utilized DCS, a novel blood flow measurement technique, to quantitatively evaluate the effect of MT on local blood flow. By adopting a diffuse optical technique with sufficient sensitivity for blood flow measurement in deep tissues (Shang et al., 2013; Yu et al., 2007), we confirmed the effect of MT in increasing the local muscular blood flow. The ∼40% increase in post-MT blood flow replicated the earlier single-case DCS study of massage therapy applied to the lower leg (Munk et al., 2012), confirming the effectiveness of manually applied physical therapy in facilitating muscular microcirculation. The simultaneous measurement of ECG and blood pressure further confirmed that MT could increase the blood flow to the targeted muscle while minimally affecting the systemic circulatory function. These results support the benefit of MT in patients who require increased muscle blood flow but are restricted from or unable to perform physical exercise due to their chronic disease states and/or frailty.

A recent study by Monteiro Rodrigues et al. (2020) reported a distinct temporal pattern of MT-related cutaneous blood flow measured using Laser Doppler flowmetry (LDF) and reflection photoplethysmography (PPG), which increased during therapy but returned to baseline immediately after cessation. The absence and presence of the post-MT increase in blood flow in the LDF/PPG and DCS, respectively, suggest the potential role of MT in selectively facilitating the circulatory activity of the targeted muscle. Although its detailed physiological mechanism is beyond the scope of this observational study, the selective increase in blood flow to the target muscle under a stable central arterial blood pressure could be explained by neuronal and metabolic regulation of peripheral microcirculation. The passive movement of skeletal muscles suppresses the muscle sympathetic nervous activity as much as active exercise (Doherty et al., 2018), which may promote local blood flow to the intervention site and the consequent decrease in other locations. However, this type of blood flow redistribution may be minor according to a previous study investigating the effect of dry needling stimulation on the blood flow changes at various regions of the trapezius muscle (Cagnie et al., 2012). Mechanical stimulation increased the blood flow at the target region in the trapezius muscle, but the blood flow at the neighboring and contralateral regions of the same muscle showed no significant changes. Rather, the mechanical stimulation could have induced shear stress-mediated vasodilation at the target tissue, thus decreasing microvascular resistance and improving the blood flow (Gregory and Mars, 2005; Nelson, 2015; Kruse et al., 2016). This hypothesis is partially supported by a recent near-infrared spectroscopy study by Soares et al. (2020), which demonstrated that a rolling massage improved the vasodilation response in the skeletal muscle without changing the endothelial function of the upstream conduit artery.

Furthermore, the trapezius muscle is a plate-like muscle that receives nourishment from multiple arteries in the trunk. In addition, its muscle fibers and veins cross diagonally, and the veins do not have valves (Nakamura et al., 2006). This unique anatomical feature of the trapezius muscle may interfere with the muscle pumping action necessary to facilitate venous return in the limbs and may cause venous blood retention. The repetitive mechanical pressure applied during MT could promote the muscle blood flow *via* passive muscle pumping and/or the corresponding vasodilation due to shear stress (Gregory and Mars, 2005; Nelson, 2015; Kruse et al., 2016).

The increase in post-CT blood flow in the trapezius muscle could be derived from the decrease in sympathetic activity innervating the skeletal muscle microvasculature due to prolonged resting in the prone position. Another cause may be the continuous exposure to near-infrared light used in DCS measurement (Ferraresi et al., 2012; Cheng et al., 2020). However, this contribution would be minor compared to that from MT.

Another important finding of this study is that the effect of MT on promoting local muscle blood flow is enhanced in muscles with stiffness. Since an increase in blood flow is positively associated with reduced muscle stiffness (Hotta et al., 2018; Caliskan et al., 2019), we first hypothesized that participants with muscle stiffness would show decreased baseline blood flow, as reported in patients with trapezius myalgia (Larsson et al., 1999) and greater recovery of blood flow after MT. However, the participants showed comparable baseline blood flow regardless of muscle stiffness, and a difference between groups was only found in post-MT muscle circulatory functions. MT significantly improved the blood flow and increased the vascular conductance selectively in participants with muscle stiffness. The local blood flow after 5 min of MT was significantly greater in participants with muscle stiffness than in those without. The lack of differences in baseline circulatory functions may be due to individual differences in the anatomical structure and/or moderate symptoms of muscle stiffness in participants who were not under clinical treatment. The higher demand for blood flow due to overstraining of the trapezius muscle in participants with muscle stiffness could be another possibility. Nevertheless, DCS could successfully capture the effect of MT on the local circulatory function in these participants, confirming its potential applicability to evaluate microcirculation in clinical settings and in sports conditioning (Shang et al., 2017). In future investigations, more objective scientific methods, such as the stiffness test or pain threshold scale, could be used to measure the improvement of shoulder stiffness symptoms in combination with the evaluation of muscle blood flow.

Local 5-min MT had little effect on systemic circulatory functions, such as blood pressure, HR, and cardiac autonomic activity. However, the effect of MT on systemic circulatory function may vary depending on the clinical status of the patient (Nelson, 2015), the form of therapy (Cambron et al., 2006; Diego and Field, 2009), and MT duration (Kaye et al., 2008). Further research on the combined systemic and local circulatory responses is required, especially regarding the clinical application of MT.

There are limitations in the interpretation of the results of this study. The participants in the group with trapezius muscle stiffness were all women. Further investigation is needed in male participants with muscle stiffness to generalize the effect of MT on increasing the blood flow of the stiff muscle. Second, the relevance of the post-MT blood flow recovery on the reduction of muscle stiffness was not evaluated since palpation and participant interviews are subjective, and a placebo effect could not be avoided. In addition, the required duration of MT for sufficient blood flow recovery could vary depending on the severity of the muscle stiffness. To avoid these potential confounding factors, we focused on the quantitative measures of muscle blood flow and systemic circulatory functions derived from a fixed MT duration. Further investigations employing emerging technologies such as ultrasound elastography (Kuo et al., 2013; Brandenburg et al., 2014; Taljanovic et al., 2017) are required to objectively evaluate the clinical effect of MT. Third, although we applied the kneading method to avoid direct mechanical stress to the skin surface of the measurement site, the cutaneous blood flow could affect the measured BFI value due to the intrinsic nature of DCS, which attaches optical probes to the skin surface (Didier et al., 2020; Bartlett et al., 2021). However, the difference in the temporal pattern of current BFI and cutaneous blood flow responses reported by Monteiro Rodrigues et al. (2020) suggests a major contribution of muscular blood flow to BFI responses. The selective increase in post-MT blood flow found in the participants with muscle stiffness also suggests that the increased BFI mainly originates from the recovered blood flow on the muscle target of the MT. The selective effect on muscles with potentially insufficient blood flow confirmed the validity of MT, which has been traditionally developed and selected by the Japanese community to ameliorate skeletal muscle strain and/or pain (Shibata et al., 2019).

In summary, our results demonstrated the usefulness of the emerging DCS technology for non-invasive and direct observation of an MT-induced blood flow increase in the local muscle. The portability of DCS enables bedside monitoring of blood flow recovery in participants with muscle stiffness, which could be further utilized in patient-oriented manual treatment and for therapists’ training. Future studies should investigate the relationship between the improvement of local muscle blood flow and physical status in patients receiving MT treatments.

## Data Availability

The datasets analyzed in this study will be made available upon request from the corresponding author.

## References

[B1] Aluwi N. I. A. B. Ono Y. Hara N. (2016). Stress Evaluation Based on Changes in the Pupillary Diameter of Human Eye. Auton. Neurosci. 201, 73. 10.1016/j.autneu.2016.09.009

[B2] Barbero M. Bertoli P. Cescon C. Macmillan F. Coutts F. Gatti R. (2012). Intra-rater Reliability of an Experienced Physiotherapist in Locating Myofascial Trigger Points in Upper Trapezius Muscle. J. Man. Manipulative Ther. 20, 171–177. 10.1179/2042618612Y.0000000010 PMC3500129 24179324

[B3] Bartlett M. F. Akins J. D. Oneglia A. P. Brothers R. M. Wilkes D. Nelson M. D. (2021). Impact of Cutaneous Blood Flow on NIR-DCS Measures of Skeletal Muscle Blood Flow index. J. Appl. Physiol. 131, 914–926. 10.1152/japplphysiol.00337.2021 34264131 PMC8526347

[B4] Boas D. A. Campbell L. E. Yodh A. G. (1995). Scattering and Imaging with Diffusing Temporal Field Correlations. Phys. Rev. Lett. 75, 1855–1858. 10.1103/PhysRevLett.75.1855 10060408

[B5] Brandenburg J. E. Eby S. F. Song P. Zhao H. Brault J. S. Chen S. (2014). Ultrasound Elastography: the New Frontier in Direct Measurement of Muscle Stiffness. Arch. Phys. Med. Rehabil. 95, 2207–2219. 10.1016/j.apmr.2014.07.007 25064780 PMC4254343

[B6] Cagnie B. Barbe T. De Ridder E. Van Oosterwijck J. Cools A. Danneels L. (2012). The Influence of Dry Needling of the Trapezius Muscle on Muscle Blood Flow and Oxygenation. J. Manipulative Physiol. Ther. 35, 685–691. 10.1016/j.jmpt.2012.10.005 23206963

[B7] Caliskan E. Akkoc O. Bayramoglu Z. Gozubuyuk O. B. Kural D. Azamat S. (2019). Effects of Static Stretching Duration on Muscle Stiffness and Blood Flow in the Rectus Femoris in Adolescents. Med. Ultrason. 21, 136–143. 10.11152/mu-1859 31063516

[B8] Cambron J. A. Dexheimer J. Coe P. (2006). Changes in Blood Pressure after Various Forms of Therapeutic Massage: a Preliminary Study. J. Altern. Complement. Med. 12, 65–70. 10.1089/acm.2006.12.65 16494570

[B9] Carp S. A. Dai G. P. Boas D. A. Franceschini M. A. Kim Y. R. (2010). Validation of Diffuse Correlation Spectroscopy Measurements of Rodent Cerebral Blood Flow with Simultaneous Arterial Spin Labeling MRI; towards MRI-Optical Continuous Cerebral Metabolic Monitoring. Biomed. Opt. Express 1, 553–565. 10.1364/BOE.1.000553 21258489 PMC3017992

[B10] Çetkin M. Bahşi İ. Orhan M. (2019). The Massage Approach of Avicenna in the Canon of Medicine. Amha 17, 103–114. 10.31952/amha.17.1.6 31315411

[B11] Cheng Y.-C. Lung C.-W. Jan Y.-K. Kuo F.-C. Lin Y.-S. Lo Y.-C. (2020). Evaluating the Far-Infrared Radiation Bioeffects on Micro Vascular Dysfunction, Nervous System, and Plantar Pressure in Diabetes Mellitus. The Int. J. Lower Extremity Wounds 19, 125–131. 10.1177/1534734619880741 31625431

[B12] Didier K. D. Hammer S. M. Alexander A. M. Caldwell J. T. Sutterfield S. L. Smith J. R. (2020). Microvascular Blood Flow during Vascular Occlusion Tests Assessed by Diffuse Correlation Spectroscopy. Exp. Physiol. 105, 201–210. 10.1113/EP087866 31713942

[B13] Diego M. A. Field T. (2009). Moderate Pressure Massage Elicits a Parasympathetic Nervous System Response. Int. J. Neurosci. 119, 630–638. 10.1080/00207450802329605 19283590

[B14] Diego M. A. Field T. Sanders C. Hernandez-Reif M. (2004). Massage Therapy of Moderate and Light Pressure and Vibrator Effects on EEG and Heart Rate. Int. J. Neurosci. 114, 31–44. 10.1080/00207450490249446 14660065

[B15] Doherty C. J. Incognito A. V. Notay K. Burns M. J. Slysz J. T. Seed J. D. (2018). Muscle Sympathetic Nerve Responses to Passive and Active One-Legged Cycling: Insights into the Contributions of central Command. Am. J. Physiology-Heart Circulatory Physiol. 314, H3–H10. 10.1152/ajpheart.00494.2017 PMC5866391 28939650

[B16] Domingo A. R. Diek M. Goble K. M. Maluf K. S. Goble D. J. Baweja H. S. (2017). Short-duration Therapeutic Massage Reduces Postural Upper Trapezius Muscle Activity. NeuroReport 28, 108–110. 10.1097/WNR.0000000000000718 27977513

[B17] Dong J. Bi R. Ho J. H. Thong P. S. P. Soo K.-C. Lee K. (2012). Diffuse Correlation Spectroscopy with a Fast Fourier Transform-Based Software Autocorrelator. J. Biomed. Opt. 17, 0970041–0997001. 10.1117/1.JBO.17.9.097004 23085922

[B18] Driessen M. T. Lin C.-W. C. van Tulder M. W. (2012). Cost-effectiveness of Conservative Treatments for Neck Pain: a Systematic Review on Economic Evaluations. Eur. Spine J. 21, 1441–1450. 10.1007/s00586-012-2272-5 22447407 PMC3535241

[B19] Drust B. Atkinson G. Gregson W. French D. Binningsley D. (2003). The Effects of Massage on Intra Muscular Temperature in the Vastus Lateralis in Humans. Int. J. Sports Med. 24, 395–399. 10.1055/s-2003-41182 12905085

[B20] Durduran T. Choe R. Baker W. B. Yodh A. G. (2010). Diffuse Optics for Tissue Monitoring and Tomography. Rep. Prog. Phys. 73, 076701. 10.1088/0034-4885/73/7/076701 26120204 PMC4482362

[B21] Englund E. K. Langham M. C. (2020). Quantitative and Dynamic MRI Measures of Peripheral Vascular Function. Front. Physiol. 11, 120. 10.3389/fphys.2020.00120 32184733 PMC7058683

[B22] Ferraresi C. Hamblin M. R. Parizotto N. A. (2012). Low-level Laser (Light) Therapy (LLLT) on Muscle Tissue: Performance, Fatigue and Repair Benefited by the Power of Light. Photon. Lasers Med 1, 267–286. 10.1515/plm-2012-0032 PMC3635110 23626925

[B23] Flodgren G. M. Hellström F. B. Fahlström M. Crenshaw A. G. (2006). Effects of 30 versus 60 Min of Low-Load Work on Intramuscular Lactate, Pyruvate, Glutamate, Prostaglandin E2 and Oxygenation in the Trapezius Muscle of Healthy Females. Eur. J. Appl. Physiol. 97, 557–565. 10.1007/s00421-006-0216-7 16767442

[B24] Goats G. C. (1994). Massage--the Scientific Basis of an Ancient Art: Part 1. The Techniques. Br. J. Sports Med. 28, 149–152. 10.1136/bjsm.28.3.149 8000809 PMC1332055

[B25] Grafton S. Mazziotta J. Presty S. Friston K. Frackowiak R. Phelps M. (1992). Functional Anatomy of Human Procedural Learning Determined with Regional Cerebral Blood Flow and PET. J. Neurosci. 12, 2542–2548. 10.1523/JNEUROSCI.12-07-02542.1992 1613546 PMC6575851

[B26] Gregory M. A. Mars M. (2005). Compressed Air Massage Causes Capillary Dilation in Untraumatised Skeletal Muscle: a Morphometric and Ultrastructural Study. Physiotherapy 91, 131–137. 10.1016/j.physio.2004.11.007

[B27] Guoqiang Yu K. G. Gurley K. Yu G. (2013). Diffuse Correlation Spectroscopy (DCS) for Assessment of Tissue Blood Flow in Skeletal Muscle: Recent Progress. Anat. Physiol. 03, 128. 10.4172/2161-0940.1000128 PMC3979478 24724043

[B28] Hinds T. McEwan I. Perkes J. Dawson E. Ball D. George K. (2004). Effects of Massage on Limb and Skin Blood Flow after Quadriceps Exercise. Med. Sci. Sports Exerc. 36, 1308–1313. 10.1249/01.mss.0000135789.47716.db 15292737

[B29] Hotta K. Behnke B. J. Arjmandi B. Ghosh P. Chen B. Brooks R. (2018). Daily Muscle Stretching Enhances Blood Flow, Endothelial Function, Capillarity, Vascular Volume and Connectivity in Aged Skeletal Muscle. J. Physiol. 596, 1903–1917. 10.1113/JP275459 29623692 PMC5978284

[B30] Ichinose M. Nakabayashi M. Ono Y. (2019). Difference in the Integrated Effects of Sympathetic Vasoconstriction and Local Vasodilation in Human Skeletal Muscle and Skin Microvasculature. Physiol. Rep. 7, e14070. 10.14814/phy2.14070 30980512 PMC6461711

[B31] Ichinose M. Nakabayashi M. Ono Y. (2021). Rapid Vasodilation within Contracted Skeletal Muscle in Humans: New Insight from Concurrent Use of Diffuse Correlation Spectroscopy and Doppler Ultrasound. Am. J. Physiology-Heart Circulatory Physiol. 320, H654–H667. 10.1152/ajpheart.00761.2020 33337963

[B32] Ichinose M. Nakabayashi M. Ono Y. (2018). Sympathoexcitation Constrains Vasodilation in the Human Skeletal Muscle Microvasculature during Postocclusive Reactive Hyperemia. Am. J. Physiology-Heart Circulatory Physiol. 315, H242–H253. 10.1152/ajpheart.00010.2018 29652542

[B33] Ishikawa H. Muraki T. Morise S. Sekiguchi Y. Yamamoto N. Itoi E. (2017). Changes in Stiffness of the Dorsal Scapular Muscles before and after Computer Work: a Comparison between Individuals with and without Neck and Shoulder Complaints. Eur. J. Appl. Physiol. 117, 179–187. 10.1007/s00421-016-3510-z 27913925

[B34] Kaye A. D. Kaye A. J. Swinford J. Baluch A. Bawcom B. A. Lambert T. J. (2008). The Effect of Deep-Tissue Massage Therapy on Blood Pressure and Heart Rate. J. Altern. Complement. Med. 14, 125–128. 10.1089/acm.2007.0665 18315516

[B35] Kitano A. Shoemaker J. K. Ichinose M. Wada H. Nishiyasu T. (2005). Comparison of Cardiovascular Responses between Lower Body Negative Pressure and Head-Up Tilt. J. Appl. Physiol. 98, 2081–2086. 10.1152/japplphysiol.00563.2004 15761089

[B36] Kramer A. Kramer K. Z. (2020). The Potential Impact of the Covid-19 Pandemic on Occupational Status, Work from home, and Occupational Mobility. J. Vocational Behav. 119, 103442. 10.1016/j.jvb.2020.103442 PMC7205621 32390661

[B37] Kruse N. T. Silette C. R. Scheuermann B. W. (2016). Influence of Passive Stretch on Muscle Blood Flow, Oxygenation and central Cardiovascular Responses in Healthy Young Males. Am. J. Physiology-Heart Circulatory Physiol. 310, H1210–H1221. 10.1152/ajpheart.00732.2015 26945077

[B38] Kuo W.-H. Jian D.-W. Wang T.-G. Wang Y.-C. (2013). Neck Muscle Stiffness Quantified by Sonoelastography Is Correlated with Body Mass index and Chronic Neck Pain Symptoms. Ultrasound Med. Biol. 39, 1356–1361. 10.1016/j.ultrasmedbio.2012.11.015 23683408

[B39] Laborde S. Mosley E. Thayer J. F. (2017). Heart Rate Variability and Cardiac Vagal Tone in Psychophysiological Research - Recommendations for experiment Planning, Data Analysis, and Data Reporting. Front. Psychol. 08, 213. 10.3389/fpsyg.2017.00213 PMC5316555 28265249

[B40] Larsson R. Öberg Å. P. Larsson S.-E. (1999). Changes of Trapezius Muscle Blood Flow and Electromyography in Chronic Neck Pain Due to Trapezius Myalgia. Pain 79, 45–50. 10.1016/S0304-3959(98)00144-4 9928775

[B41] Lin C.-C. Hua S.-H. Lin C.-L. Cheng C.-H. Liao J.-C. Lin C.-F. (2020). Impact of Prolonged Tablet Computer Usage with Head Forward and Neck Flexion Posture on Pain Intensity, Cervical Joint Position Sense and Balance Control in Mechanical Neck Pain Subjects. J. Med. Biol. Eng. 40, 372–382. 10.1007/s40846-020-00525-8

[B42] Monteiro Rodrigues L. Rocha C. Ferreira H. T. Silva H. N. (2020). Lower Limb Massage in Humans Increases Local Perfusion and Impacts Systemic Hemodynamics. J. Appl. Physiol. 128, 1217–1226. 10.1152/japplphysiol.00437.2019 32191595

[B43] Munk N. Symons B. Shang Y. Cheng R. Yu G. (2012). Noninvasively Measuring the Hemodynamic Effects of Massage on Skeletal Muscle: a Novel Hybrid Near-Infrared Diffuse Optical Instrument. J. Bodywork Mov. Therapies 16, 22–28. 10.1016/j.jbmt.2011.01.018 22196423

[B44] Nakabayashi M. Ono Y. (2017). Detection of Blood Flow Speed in Shallow and Deep Tissues Using Diffuse Correlation Spectroscopy. Abe 6, 53–58. 10.14326/abe.6.53

[B45] Nakabayashi M. Ono Y. Ichinose M. (2018). “Evaluation of Blood Flow in Human Exercising Muscle by Diffuse Correlation Spectroscopy: a Phantom Model Study,” in Design and Quality for Biomedical Technologies XI. Editors Raghavachari R. Liang R. Pfefer T. J.. 10.1117/12.2288044

[B46] Nakamura T. Murakami G. Noriyasu S. Yoshio M. Sato I. Uchiyama E. (2006). Morphometrical Study of Arteries and Veins in the Human Sheet-like Muscles (Pectoralis Major, Latissimus Dorsi, Gluteus Maximus and Trapezius) with Special Reference to a Paradoxical Venous Merging Pattern of the Trapezius. Ann. Anat. - Anatomischer Anzeiger 188, 243–253. 10.1016/j.aanat.2005.11.011 16711163

[B47] Nelson N. L. (2015). Massage Therapy: Understanding the Mechanisms of Action on Blood Pressure. A Scoping Review. J. Am. Soc. Hypertens. 9, 785–793. 10.1016/j.jash.2015.07.009 26324746

[B48] Nishikitani M. Inoue S. Yano E. (2008). Competition or Complement: Relationship between Judo Therapists and Physicians for Elderly Patients with Musculoskeletal Disease. Environ. Health Prev. Med. 13, 123–129. 10.1007/s12199-007-0021-x 19568896 PMC2698256

[B49] Ono Y. Esaki K. Takahashi Y. Nakabayashi M. Ichinose M. Lee K. (2018). Muscular Blood Flow Responses as an Early Predictor of the Severity of Diabetic Neuropathy at a Later Stage in Streptozotocin-Induced Type I Diabetic Rats: a Diffuse Correlation Spectroscopy Study. Biomed. Opt. Express 9, 4539–4551. 10.1364/BOE.9.004539 30615744 PMC6157794

[B50] Patterson M. S. Andersson-Engels S. Wilson B. C. Osei E. K. (1995). Absorption Spectroscopy in Tissue-Simulating Materials: a Theoretical and Experimental Study of Photon Paths. Appl. Opt. 34, 22–30. 10.1364/AO.34.000022 20963080

[B51] Portillo-Soto A. Eberman L. E. Demchak T. J. Peebles C. (2014). Comparison of Blood Flow Changes with Soft Tissue Mobilization and Massage Therapy. J. Altern. Complement. Med. 20, 932–936. 10.1089/acm.2014.0160 25420037

[B52] Reuter D. A. Huang C. Edrich T. Shernan S. K. Eltzschig H. K. (2010). Cardiac Output Monitoring Using Indicator-Dilution Techniques: Basics, Limits, and Perspectives. Anesth. Analg. 110, 799–811. 10.1213/ANE.0b013e3181cc885a 20185659

[B53] Saltin B. Rådegran G. Koskolou M. D. Roach R. C. (1998). Skeletal Muscle Blood Flow in Humans and its Regulation during Exercise. Acta Physiol. Scand. 162, 421–436. 10.1046/j.1365-201X.1998.0293e.x 9578388

[B54] Sandberg M. L. Sandberg M. K. Dahl J. (2007). Blood Flow Changes in the Trapezius Muscle and Overlying Skin Following Transcutaneous Electrical Nerve Stimulation. Phys. Ther. 87, 1047–1055. 10.2522/ptj.20060178 17578938

[B55] Sciotti V. M. Mittak V. L. DiMarco L. Ford L. M. Plezbert J. Santipadri E. (2001). Clinical Precision of Myofascial Trigger point Location in the Trapezius Muscle. Pain 93, 259–266. 10.1016/S0304-3959(01)00325-6 11514085

[B56] Sefton J. M. Yarar C. Berry J. W. Pascoe D. D. (2010). Therapeutic Massage of the Neck and Shoulders Produces Changes in Peripheral Blood Flow when Assessed with Dynamic Infrared Thermography. J. Altern. Complement. Med. 16, 723–732. 10.1089/acm.2009.0441 20590481

[B57] Shang Y. Li T. Yu G. (2017). Clinical Applications of Near-Infrared Diffuse Correlation Spectroscopy and Tomography for Tissue Blood Flow Monitoring and Imaging. Physiol. Meas. 38, R1–R26. 10.1088/1361-6579/aa60b7 28199219 PMC5726862

[B58] Shibata Y. Nakamura M. Nakamura H. Okada E. Ojima T. (2019). Coping Behaviors for Skeletal Muscle Injuries and Disorders Among Community-Dwelling Elderly Persons in Japan. J. Phys. Ther. Sci. 31, 536–539. 10.1589/jpts.31.536 31417217 PMC6642893

[B59] Shoemaker J. K. Tiidus P. M. Mader R. (1997). Failure of Manual Massage to Alter Limb Blood Flow: Measures by Doppler Ultrasound. Med. Sci. Sports Exerc. 29, 610–614. 10.1097/00005768-199705000-00004 9140896

[B60] Soares R. N. Inglis E. C. Khoshreza R. Murias J. M. Aboodarda S. J. (2020). Rolling Massage Acutely Improves Skeletal Muscle Oxygenation and Parameters Associated with Microvascular Reactivity: the First Evidence-Based Study. Microvasc. Res. 132, 104063. 10.1016/j.mvr.2020.104063 32841627

[B61] Taljanovic M. S. Gimber L. H. Becker G. W. Latt L. D. Klauser A. S. Melville D. M. (2017). Shear-Wave Elastography: Basic Physics and Musculoskeletal Applications. RadioGraphics 37, 855–870. 10.1148/rg.2017160116 28493799 PMC5452887

[B62] Task Force of the European Society of Cardiology, and the North American Society of Pacing and Electrophysiology (1996). Heart Rate Variability: Standards of Measurement, Physiological Interpretation and Clinical Use. Task Force of the European Society of Cardiology and the North American Society of Pacing and Electrophysiology. Circulation 93, 1043–1065. 10.1161/01.CIR.93.5.1043 8598068

[B63] Thomas D. J. Zilkha E. Redmond S. Du Boulay G. H. Marshall J. Ross Russell R. W. (1979). An Intravenous ^133^xenon Clearance Technique for Measuring Cerebral Blood Flow. J. Neurol. Sci. 40, 53–63. 10.1016/0022-510X(79)90008-X 762594

[B64] Tiidus P. (1997). Manual Massage and Recovery of Muscle Function Following Exercise: a Literature Review. J. Orthop. Sports Phys. Ther. 25, 107–112. 10.2519/jospt.1997.25.2.107 9007768

[B65] Tiidus P. M. Shoemaker J. K. (1995). Effleurage Massage, Muscle Blood Flow and Long-Term post-exercise Strength Recovery. Int. J. Sports Med. 16, 478–483. 10.1055/s-2007-973041 8550258

[B66] Wigmore D. M. Damon B. M. Pober D. M. Kent-Braun J. A. (2004). MRI Measures of Perfusion-Related Changes in Human Skeletal Muscle during Progressive Contractions. J. Appl. Physiol. 97, 2385–2394. 10.1152/japplphysiol.01390.2003 15298991

[B67] World Health Organization (2001). Geneva: Programme on Traditional MedicineWorld Health Organization. Available at: https://apps.who.int/iris/handle/10665/42452 .Legal Status of Traditional Medicine and Complementary/alternative Medicine: a Worldwide Review

[B68] Yu G. (2012). Diffuse Correlation Spectroscopy (DCS): a Diagnostic Tool for Assessing Tissue Blood Flow in Vascular-Related Diseases and Therapies. Cmir 8, 194–210. 10.2174/157340512803759875

[B69] Yu G. Durduran T. Lech G. Zhou C. Chance B. Mohler E. R. (2005). Time-dependent Blood Flow and Oxygenation in Human Skeletal Muscles Measured with Noninvasive Near-Infrared Diffuse Optical Spectroscopies. J. Biomed. Opt. 10, 024027. 10.1117/1.1884603 15910100

[B70] Yu G. Floyd T. F. Durduran T. Zhou C. Wang J. Detre J. A. (2007). Validation of Diffuse Correlation Spectroscopy for Muscle Blood Flow with Concurrent Arterial Spin Labeled Perfusion MRI. Opt. Express 15, 1064–1075. 10.1364/OE.15.001064 19532334

[B71] Zhou C. Eucker S. A. Durduran T. Yu G. Ralston J. Friess S. H. (2009). Diffuse Optical Monitoring of Hemodynamic Changes in Piglet Brain with Closed Head Injury. J. Biomed. Opt. 14, 034015. 10.1117/1.3146814 19566308 PMC3169814

